# Cultural Diversity Impacts Caregiving Experiences: A Comprehensive Exploration of Differences in Caregiver Burdens, Needs, and Outcomes

**DOI:** 10.7759/cureus.46537

**Published:** 2023-10-05

**Authors:** Jessica T Tran, Bunnarin Theng, Huey-Ming Tzeng, Mukaila Raji, Hani Serag, Miaolung Shih, Wei-Chen (Miso) Lee

**Affiliations:** 1 John Sealy School of Medicine, University of Texas Medical Branch, Galveston, USA; 2 School of Nursing, University of Texas Medical Branch, Galveston, USA; 3 Department of Internal Medicine, Division of Geriatrics and Palliative Medicine, University of Texas Medical Branch, Galveston, USA; 4 Department of Internal Medicine, Division of Endocrinology, University of Texas Medical Branch, Galveston, USA; 5 Humanistic Buddhism Practice (HBP), Osher Lifelong Learning Institute at University of Texas Medical Branch, Galveston, USA; 6 Department of Family Medicine, University of Texas Medical Branch, Galveston, USA

**Keywords:** cultural values, culture, ethnicity, race, resources, challenges, caregiver, caregiving burden

## Abstract

Background

Many Americans are informal caregivers providing unpaid care for their loved ones living with chronic conditions, such as dementia and heart failure. As the US population continues to age and live longer with more complex chronic conditions, informal caregivers play an increasingly important role in the care of older adults with functional impairment and multiple comorbidities. Caregivers face many challenges in managing the health of themselves and their loved ones, including physical, emotional, and financial burdens, which may potentially vary by race and ethnicity. Therefore, it is critical to develop culturally tailored solutions, such as smart technology, aimed at improving the quality of life of informal caregivers and care recipients from diverse backgrounds.

Methods

Data were collected from a convenient sample of 69 informal caregivers in Texas who were members or volunteers for either the International Buddhist Progress Society-Dallas (IBPS Dallas) or University of Texas Medical Branch (UTMB). Caregivers answered questions about their caregiving experiences, including the type of care they provided, challenges they faced, and lessons learned. Responses were stratified by race/ethnicity (White, Hispanic, or Asian American) to assess for potential cultural differences in caregiving experiences. A chi-squared test and one-way analysis of variance (ANOVA) were conducted.

Results

White, Hispanic, and Asian American caregivers all reported high non-medical related needs. White, Hispanic, and Asian American care recipients all had a high degree of neurological disease and functional impairment. White and Hispanic caregivers were also more likely to offer emotional support (*p*=0.007) and financial support (*p*=0.025) than Asian American caregivers. Asian American caregivers reported greater worry about the health-related knowledge of their family members (*p*=0.040) than White and Hispanic caregivers. Hispanic (18.8%) and Asian American caregivers (12.5%) reported the least knowledge of caregiving-related government policies than White caregivers (43.2%) (*p*=0.025). Hispanic (18.8%) and Asian American caregivers (18.8%) also reported the least knowledge of available support programs and services for care recipients (*p*=0.001).

Conclusions

White, Hispanic, and Asian American informal caregivers vary in their types of worries, care provided, and challenges faced. Our study found that Asian American caregivers reported greater worry about the health-related knowledge of their family members than White and Hispanic caregivers. White caregivers were better at navigating government resources and caregiver support programs than Hispanic and Asian American caregivers. While race and ethnicity are potential factors for these observed differences, several other factors may have played a role, including age, gender, income, education, patient diagnosis, and disease severity. Future research should consider these factors and evaluate a larger and more diverse sample for more definitive racial and ethnic comparisons. Understanding disparities in caregiving experiences is a critical initial step to developing culturally appropriate interventions to reduce caregiving burden and promote the health and well-being of both patients and their informal caregivers from diverse backgrounds.

## Introduction

The number of adults aged 65 and older in the United States has steadily grown since the 1960s, increasing by nearly 40% from 2010 to 2020. In 2020, 55.7 million people were 65 years old and over, accounting for nearly one in six people in the United States. By 2040, this number is projected to climb to 80.8 million [1]. Along with this increase in the older population comes an increase in the prevalence of disabling chronic conditions, such as heart failure, cancer, and Alzheimer’s disease and related dementias (ADRD), subsequently increasing the number of individuals requiring care by a family member. In 2020, 18% of adults aged 65 and older reported decreased functioning across several domains, primarily mobility, hearing, and cognition. With more adults growing older with multiple chronic conditions along with people with disabilities living longer, informal caregivers play an increasingly critical role in the care of older Americans needing assistance with activities of daily living (ADLs).

Informal caregivers are unpaid individuals, typically a spouse or adult child, who provide long-term care for their loved ones. An estimated 53 million Americans, or one in five US adults, provide unpaid care to a loved one [2]. The value of services provided by informal caregivers was estimated to be $470 billion in 2013 [3]. In addition to this heavy financial toll, caregiving places an enormous emotional, physical, and psychological burden on caregivers. Caregivers support their loved ones’ quality of life in a variety of ways, from helping them perform basic ADLs (e.g., bathing, toileting, and dressing) to managing their finances and physician visits. On average, caregivers spend 24 hours per week providing care, and caregivers who live with their recipients provide an average of 37 hours of care per week [2]. Caregivers often neglect their own health at the expense of their loved ones. In 2020, over half of caregivers in the United States were aged 50 and older, and 61% were employed while caregiving. Caregivers who reported poorer self-rated health had higher intensity or demanding care situations, greater hours worked weekly, and higher physical, financial, and emotional stress [2]. Therefore, it is important for healthcare professionals, public health experts, and policymakers to develop and implement solutions to reduce caregiving burden and support the health and well-being of both caregivers and care recipients.

Not only is the US population growing older, but it is also becoming more diverse. Racial and ethnic minority populations make up a considerable proportion of the aging population and are anticipated to grow at a faster rate than the White population [1]. In 2020, 24% of persons aged 65 and older were part of a racial and ethnic minority group. By 2060, nearly half of older adults are projected to be racial/ethnic minorities [4]. This diversity is also evident among caregivers, with 39% of adult caregivers in the United States identifying as a racial and ethnic minority. Hispanic caregivers have the highest reported prevalence of informal caregiving (21%), followed by Asian American (19.7%) and White (16.9%) [3]. In order to develop appropriate interventions supporting the health and quality of life of caregivers and care recipients from diverse backgrounds, it is crucial to understand how caregiving experiences may vary by race and ethnicity.

Our study aims to understand how racial and cultural differences between White, Hispanic, and Asian American caregivers may impact their needs, motivations, burdens, coping strategies, and self-rated health and growth. We administered a survey in different cities across Texas to gather information on caregivers’ experiences, including their challenges and lessons learned, and performed a content analysis of their responses. We intend to use this analysis to guide our design of a culturally sensitive app, *utmbHealthyBrain,* that can best support caregivers’ needs. Given the high rate of multigenerational living among Hispanics [5] and a strong sense of filial obligation among Asian Americans [6], we expect that Hispanics and Asian Americans will have greater caregiving-related needs than the White population and thus report greater burden [7-9]. Hispanics and Asian Americans also face greater obstacles to receiving caregiving support due to cultural and linguistic barriers and likely have less knowledge of available support programs and services [10]. However, due to the heavy emphasis on collectivism in both Latino and Asian cultures, in contrast to the individualism of the Western society, we expect Hispanic and Asian American caregivers to report more positive changes in relationships with their care recipients and lessons learned than White caregivers [11,12].

## Materials and methods

Study design

Technologies, such as smartphones and wearable electronics, can assist users with certain daily functions, such as sending reminders to take medications. However, simply utilizing smartphones cannot facilitate interpersonal relationships that are critical for elders’ and caregivers’ quality of life and mental well-being. The Institute for Healthcare Improvement (IHI) has developed a 4M (what matters most to patients, medication, mentation, and mobility) model, which encourages healthy systems to become age-friendly by collaborating with older adults and caregivers [13]. The new trend for age-friendly initiatives is focused on decentralizing healthy systems and transforming a provider-centered to patient-centered care model with interventions and programs targeted to the 4Ms [14].

In light of this trend, an academic-community partnership was formed between the International Buddhist Progress Society-Dallas (IBPS Dallas) and the University of Texas Medical Branch (UTMB). The goal of this partnership was to conduct a caregiver survey to collect information on their caregiving experiences, both challenges and lessons learned, to better design an app and AI that can meet caregivers’ needs. A convenient sample was formed to include survey respondents who were members or volunteers for either IBPS Dallas or UTMB Osher Lifelong Learning Institute (OLLI). The project team formed this convenient sample to consult laypeople on their advice on designing a smartphone app via an anonymous survey. As such, the study is not considered a human subject research, and no IRB approval was required for this study.

Study sample

Affiliated members or volunteers who mentioned that they are caregivers of any individual aged 50 years and older were invited to participate in this survey. The exclusion criteria were (1) caregivers who are younger than 18, (2) who did not look after any family members aged 50 years and older, or (3) who is not able to read and write in English. We invited volunteers who took care of adults aged 50 and older to fill out a survey, considering the 50+ age standard at UTMB OLLI. We excluded duplicate responses, those who were paid for caregiving, and those who cared for a client/patient. The remaining sample consists of informal family caregivers. Over three months in 2022, the project team collected 84 responses, two of which were excluded because of duplicated submissions. All volunteers or members were based in Dallas, Houston, and Galveston areas in Texas at that time, which might limit the generalizability of this study to other populations.

Measurements

This is a mixed-method study that collected both quantitative and qualitative information on caregivers’ experiences. The primary outcomes of this study are threefold: (1) quantitative (frequency and percentage of type of care reported by survey respondents, such as the percentage of caregivers who self-reported that they provided medical support to their care recipients), (2) quantitative (mean and standard errors for the questions about each kind of worry as a caregiver), and (3) qualitative (survey questions about caregiving challenges, such as financial burden, and lessons learned, such as the change in relationship between care recipients and caregivers). Responses to the type of care were coded as yes (1) or no (0). Responses to the type of worry were coded from not worried at all (1) to worried a lot (5).

The independent variable of this study is the caregiver's race and ethnicity category. Among 82 valid responses, three categories (African Americans, Native Americans, and multiple races) had numbers less than 10 and would not yield meaningful results. The final sample consisted of 38 White, 16 Asian American, and 16 Hispanic informal caregivers. Given that one of the respondents was a paid caregiver, we also excluded her in the qualitative analysis (total: 69 responses) to ensure that all the findings are relevant to informal, unpaid caregivers.

Analysis

Caregivers' characteristics (e.g., age), type of care (e.g., emotional support), and worry (e.g., lack of medical knowledge) as a caregiver were summarized. A chi-squared test was performed for categorical variables, such as gender, and analysis of variance (ANOVA) test was performed for continuous variables, such as age. Fisher's exact test was also performed for cells less than 5. Any p-value less than 0.05 was considered as statistically significant. All data analyses were conducted by Stata Statistical Software, release 18 (StataCorp., 2023, College Station, TX: StataCorp LLC).

Qualitative responses were first transcribed and coded by J.T. and then cross-validated by B.T. and W.-C. L. Following the content analysis, the project team developed a list of emerging themes and recategorized each response by appropriate themes. The frequency of responses for each theme was further calculated to identify the most frequent challenges and lessons learned for caregivers. The process of recategorization was needed to reduce the redundancy as several respondents indicated their unmet needs across a series of questions. It also provided a better insight into where and how race and ethnicity contributed to different caregiving experiences.

## Results

Table 1 shows that a total of 70 caregivers self-reported their race and ethnicity as White, Hispanic, or Asian American. The comparison across the three groups shows that both White and Asian American caregivers were more likely to have received higher education (≥50.0%, *p*=0.010) than Hispanics. Both White and Hispanic caregivers were more likely to still work on a full-time job (≥75.0%, *p*=0.014), have religious beliefs in Christianity or Catholicism (*p*<0.001), have computers (*p*=0.024), and have a vision insurance plan (*p*=0.036) than Asian American caregivers. However, the Asian American respondents are more likely to speak more than two languages (mean=2.2, *p*<0.001).

**Table 1:** Individual characteristics of caregivers by race and ethnicity Note: This table contains 70 original responses. * The difference between White and Hispanic caregivers is statistically significant. @ The difference between White and Asian American caregivers is statistically significant. ‡ The difference between Hispanic and Asian American caregivers is statistically significant.

	Total (N=70)	White (n=38)	Hispanic (n=16)	Asian American (n=16)	p-value
Age (min, max)	(30, 80)	(42, 80)	(37, 69)	(30, 70)	
Age (mean, std.)*^‡^	57.1 (1.3)	59.1 (1.7)	50.1 (2.5)	59.5 (2.9)	0.641
Female (n, %)	64 (91.4%)	34 (89.5%)	16 (100.0%)	14 (87.5%)	0.532
Marriage					0.052
Single or never married	10 (14.3%)	2 (5.3%)	5 (31.3%)	3 (18.8%)	
Separated, divorced, widowed	19 (27.1%)	12 (31.6%)	5 (31.3%)	2 (12.5%)	
Married or living with a partner	41 (58.6%)	24 (63.2%)	6 (37.5%)	11 (68.8%)	
Education^‡^					0.010
High school graduate	2 (2.9%)	0 (0.0%)	2 (12.5%)	0 (0.0%)	
Associate or some college	19 (27.1%)	7 (18.4%)	9 (56.3%)	3 (18.8%)	
College degree	17 (24.3%)	11 (29.0%)	1 (6.3%)	5 (31.3%)	
Graduate degree or above	32 (45.7%)	20 (52.6%)	4 (25.0%)	8 (50.0%)	
Employment^@^^‡^					0.014
Full time	46 (65.7%)	29 (76.3%)	12 (75.0%)	5 (31.3%)	
Part time	3 (4.3%)	2 (5.3%)	1 (6.3%)	0 (0.0%)	
Retired	15 (21.4%)	5 (13.2%)	2 (12.5%)	8 (50.0%)	
Self-own business	3 (4.3%)	1 (2.6%)	1 (6.3%)	1 (6.3%)	
Not working	3 (4.3%)	1 (2.6%)	0 (0.0%)	2 (12.5%)	
Religion^@^					<0.001
Christian or Catholic	36 (51.4%)	23 (60.5%)	13 (81.3%)	0 (0.0%)	
Others	34 (48.6%)	15 (39.5%)	3 (18.8%)	16 (100.0%)	
No. of languages*^@^^‡^	1.5 (0.09)	1.2 (0.11)	1.8 (0.21)	2.2 (0.10)	<0.001
Device ownership (multiple choices)					
Smartphone	68 (97.1%)	36 (94.7%)	16 (100.0%)	16 (100.0%)	1.000
Computer^@^	62 (88.6%)	37 (97.4%)	13 (81.3%)	12 (75.0%)	0.024
Tablet	46 (65.7%)	25 (65.8%)	11 (68.8%)	10 (62.5%)	1.000
Smart home appliance	16 (22.9%)	10 (26.3%)	3 (18.8%)	3 (18.8%)	0.801
No. of devices (mean, std. deviation)*^ @^	2.7 (0.09)	2.8 (0.12)	2.7 (0.20)	2.6 (0.22)	0.224
Health insurance type (multiple choices)					
Medicare	22 (31.4%)	14 (36.8%)	3 (18.8%)	5 (31.3%)	0.462
Medicaid or other government insurance	3 (4.3%)	1 (2.6%)	1 (6.3%)	1 (6.3%)	0.589
Private insurance	53 (75.7%)	30 (79.0%)	14 (87.5%)	9 (56.3%)	0.116
Dental insurance	38 (54.3%)	22 (57.9%)	11 (68.8%)	5 (31.3%)	0.085
Vision insurance^@^	33 (47.1%)	21 (55.3%)	9 (56.3%)	3 (18.8%)	0.036
VA or military	1 (1.4%)	0 (0.0%)	1 (6.3%)	0 (0.0%)	0.457
Long-term care insurance	6 (8.6%)	2 (5.3%)	3 (18.8%)	1 (6.3%)	0.274
None	2 (2.9%)	0 (0.0%)	0 (0.0%)	2 (12.5%)	0.099
No. of driving days (min, max)	(0, 7)	(2, 7)	(3, 7)	(0, 7)	
No. of driving days (mean, std. deviation)^ @^	5.8 (0.20)	6.0 (0.27)	6.1 (0.36)	5.2 (0.50)	0.181

The comparison between two groups shows that the Hispanic (H) caregivers are significantly younger and less educated than the White (W) caregivers (H-W *p*<0.05) and Asian American (A) caregivers (H-A *p*<0.05). Next, the White caregivers significantly have more devices than the Hispanic (W-H *p*=0.0272) and Asian American caregivers (W-A *p*=0.0001). Finally, more Asian American caregivers are retired than White (A-W *p*=0.004) and Hispanic caregivers (A-H *p*=0.018). 

After excluding the respondent who self-reported as a paid caregiver, the total number of participants for the second analysis is 69 (see Table 2). Table 2 demonstrates that, compared to Asian American caregivers, White and Hispanic caregivers are more likely to offer emotional support (*p*=0.007) and financial support (*p*=0.025) to their care recipients. Because the previous analysis (Table 1) indicates that most Asian American caregivers are retired, it might explain why they were less likely to offer financial support to their family members. By comparing each of two racial groups, we also found that White caregivers are more likely to offer emotional (*p*=0.007) and financial (*p*=0.012) support than Asian American caregivers.

**Table 2:** Types of care and worries related to caregiving by race and ethnicity Note: This table excludes one person (#47) who is paid to offer formal services for clients and hence is bound by a contract, not by willingness. * A chi-squared analysis was used to compare the number of respondents with each type of support offered and worry. @ The difference between White and Asian American caregivers is statistically significant. ‡ The difference between Hispanic and Asian American caregivers is statistically significant.

	Total (N=69)	White (n=37)	Hispanic (n=16)	Asian American (n=16)	p-value*
Support you offer (n, %)					
Medical support	56 (81.2%)	32 (86.5%)	14 (87.5%)	10 (62.5%)	0.126
Functional support	30 (43.5%)	13 (35.1%)	7 (43.8%)	10 (62.5%)	0.170
Daily activities	46 (66.7%)	23 (62.2%)	14 (87.5%)	9 (56.3%)	0.128
Emotional support^@^	60 (87.0%)	35 (94.6%)	15 (93.8%)	10 (62.5%)	0.007
Financial support^@^	43 (62.3%)	28 (75.7%)	9 (56.3%)	6 (37.5%)	0.025
What worries you (mean, std. deviation)					
My knowledge^@^^‡^	2.8 (0.17)	2.6 (0.23)	2.4 (0.24)	3.6 (0.36)	0.040
My time^@^	3.6 (0.16)	3.8 (0.21)	3.6 (0.27)	2.8 (0.40)	0.015
My finance^@^^‡^	3.1 (0.19)	3.4 (0.26)	3.7 (0.37)	1.8 (0.31)	0.003
My own health^@^^‡^	3.1 (0.16)	3.3 (0.20)	3.4 (0.31)	2.3 (0.31)	0.021
Other help^@^	3.1 (0.17)	3.5 (0.21)	2.9 (0.36)	2.3 (0.33)	0.003
They accept	2.0 (0.16)	2.2 (0.22)	1.9 (0.31)	1.9 (0.31)	0.407
They appreciate	1.8 (0.14)	1.9 (0.16)	1.5 (0.27)	2.1 (0.37)	0.765
Their knowledge	3.5 (0.15)	3.4 (0.21)	3.3 (0.33)	3.8 (0.26)	0.382
Their finance	2.8 (0.19)	3.0 (0.25)	2.9 (0.43)	2.3 (0.39)	0.153
Their health	3.9 (0.17)	3.9 (0.22)	4.1 (0.37)	3.8 (0.37)	0.931
Their safety	3.7 (0.16)	3.9 (0.20)	3.8 (0.36)	3.3 (0.35)	0.177
Their adjustment	3.5 (0.19)	3.6 (0.24)	3.5 (0.4)	3.4 (0.44)	0.731

Of 12 kinds of worries, both the White and Hispanic respondents were more worried about the health and safety of their care recipients (mean>3.9) and less worried about whether their family members appreciate them (mean<1.9). On the other hand, the Asian American respondents were more worried about the health-related knowledge of their family members (mean=3.8) and less worried about their own financial ability to care for their family members (mean=1.8). We did not find any statistically significant difference in other types of worries between each of the two racial groups.

Based on these results, it seems that race and ethnicity may play a role in the observed differences in the types of worries and support provided. However, other avenues may have contributed to these differences, such as age, gender, income, education, patient diagnosis, and severity.

Content analysis results

**Figure 1:**
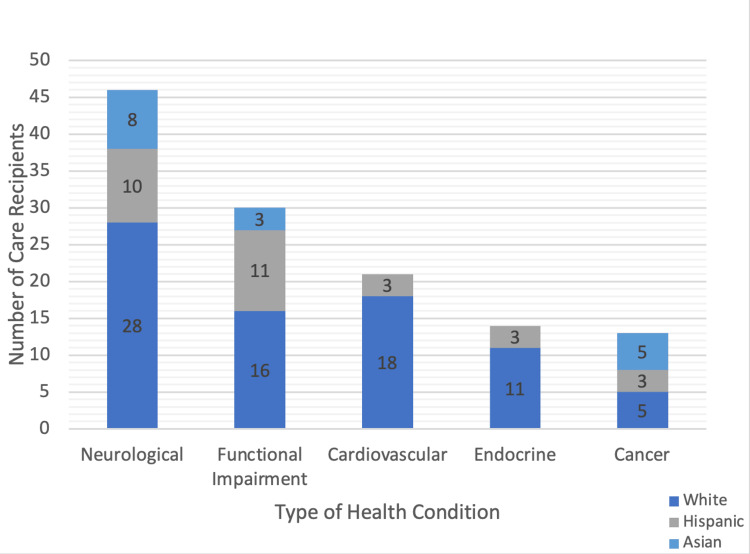
Common health conditions among care recipients by race and ethnicity

The survey asked caregivers (N=69; 37 White, 16 Hispanic, 16 Asian American) to describe the health conditions of their care recipients. Figure 1 demonstrates that the most prevalent health conditions among the care recipients were neurological disease, followed by functional impairment. Neurological disease encompasses diseases, such as Parkinson’s and Alzheimer’s diseases; functional impairment refers to limited mobility secondary to causes, such as old age or stroke. Since care recipients across all the three racial and ethnic groups had similar types of health conditions, we can rule out how the type of health condition might affect the degree or type of caregiver burden.

Over three-fourths of the White care recipients (28 out of 37; 76%) had cardiovascular diseases, such as hypertension. Endocrine diseases, such as diabetes mellitus, were also common among the White (11 out of 37; 30%) and Hispanic respondents (three out of 16; 19%). Cancer was the most prevalent health condition among the Asian American care recipients (five out of 16; 31%) compared to the White (five out of 37; 14%) and Hispanic care recipients (three out of 16; 19%).

Two overarching themes were identified based on the caregiver survey responses: (1) resources needed to alleviate burden and (2) lessons learned. A breakdown of the thematic analysis is summarized below.

Caregiver burden

In order to evaluate the resource needs of caregivers, the survey respondents were asked about the support they need to alleviate financial burden, boost their own physical and mental health, and improve their loved ones’ quality of life. Upon analysis of their responses, two main themes emerged: (1) medical-related needs (63.7%) and (2) non-medical-related needs (94.2%) (Table 3). Table 3 demonstrates that compared to the White caregivers (51.4%), Hispanic (75.0%) and Asian American caregivers (81.3%) have more medical-related needs (*p*=0.065); however, this difference was not statistically significant. White, Hispanic, and Asian American caregivers all had a similar level of non-medical-related needs (*p*=0.988). This indicates that all caregivers, regardless of race and ethnicity, desire non-medical-related support, such as additional time off, entertainment and companionship for their care recipients, and assistance with basic needs (e.g., housing, utilities, and food).

**Table 3:** Resources needed for caregiving by race and ethnicity * A chi-squared analysis was used to compare the number of respondents with each type of resource need.

Types of resources	Total (N=69)	White (n=37)	Hispanic (n=16)	Asian American (n=16)	p-value*
Medical related	44 (63.8%)	19 (51.4%)	12 (75.0%)	13 (81.3%)	0.065
Non-medical related	65 (94.2%)	35 (94.6%)	15 (93.8%)	15 (93.8%)	0.988

Medical-Related Needs

Of the 69 informal caregivers, 44 (63.8%) reported having medical-related needs. Many caregivers struggled with affording medications and desired improved insurance coverage (21.7%).

“I need more help during working hours, more money for their meds, money to help them have a life instead of just existing.”* (Participant 78, White)*

In addition, over one-third of the participants (24 out of 69) desired additional physical therapy for their care recipients and time off to exercise to maintain their own physical health. Meanwhile, 11.6% of the participants (eight out of 69) desired psychological counseling and time off for spiritual activities, such as meditation, to support their mental health. They also desired mental support for their loved ones.

“[I want to] lessen their pain and let them feel better.”* (Participant 79, Asian American)*

In addition, nearly one-third of the participants (19 out of 69) wished they had greater medical knowledge of their loved ones’ health conditions to better care for them. Interestingly, 37.5% of the Asian American respondents desired “more knowledge of the sickness” (Participant 71), compared to just 21.6% of the White respondents and 25% of the Hispanic respondents.

Non-Medical-Related Needs

Nearly all caregivers across all the three ethnic groups expressed a need for non-medical-related resources (94.2%). Approximately 13% of the respondents desired assistance with basic needs, such as housing and utilities. Nearly half of the respondents (43.5%) desired additional caregiving support and time off to rest or go on a vacation.

“I would like to have time to go to the gym, my doctor’s appointments, or go on vacation.” *(Participant 39, Hispanic)*

Nearly one-fourth of the respondents (23.2%) desired additional social activities and companionship for their loved ones. Another 10.4% of the respondents wished they lived closer to healthcare facilities to be better able to manage their loved ones’ health conditions. Other miscellaneous needs that caregivers mentioned would improve their loved ones’ quality of life included pain management, dental hygiene, transportation, and hearing aids. Interestingly, only the Asian American respondents mentioned religion and prayer as resources they need to support the well-being of themselves and their loved ones.

Lessons learned

In order to evaluate the outcomes of caregiving, the survey respondents were asked about the knowledge they have gained throughout their caregiving journey. Their responses revealed four main themes: (1) change in knowledge, care, and skills (78.3%); (2) change in personality and relationship (55.1%); (3) government policy and social norm challenges (30.4%); and (4) knowledge about programs, services, and their limitations (39.1%) (see Table 4). Table 4 demonstrates the outcomes of caregiving by race and ethnicity. The White respondents reported the most change in knowledge, care, and skills throughout their years of caregiving (86.5%). Meanwhile, the Hispanic respondents reported the most change in personality and relationship with their care recipients after taking on their caregiving role compared to before (68.8%). These differences were not significant. The Asian American (12.5%) and Hispanic respondents (18.8%) both reported having the least knowledge of the government’s role in supporting caregivers, including policies and societal norms (*p*=0.025). The Asian American and Hispanic respondents also had the least knowledge about available resources, support programs, and their limitations (*p*=0.001).

**Table 4:** Outcomes of caregiving by race and ethnicity * A chi-squared analysis was used to compare the number of respondents with each type of caregiving outcome. * The difference between the White and Hispanic caregivers is statistically significant. @ The difference between the White and Asian American caregivers is statistically significant.

Types of outcomes	Total (N=69)	White (n=37)	Hispanic (n=16)	Asian (n=16)	p-value*
Change in knowledge, care, and skills	54 (78.3%)	32 (86.5%)	12 (75.0%)	10 (62.5%)	0.128
Change in personality and relationship	38 (55.1%)	22 (59.5%)	11 (68.8%)	5 (31.3%)	0.075
Government policy and norm challenges	21 (30.4%)	16 (43.2%)	3 (18.8%)	2 (12.5%)	0.025
Knowledge about programs and services^*@^	27 (39.1%)	21 (56.8%)	3 (18.8%)	3 (18.8%)	0.001

Change in Knowledge, Care, and Skills

Over three-fourths of the caregivers (78.3%) gained valuable insights about themselves and their loved ones throughout their caregiving experience. The caregivers expressed that taking care of their loved ones enhanced their understanding of the impacts of aging on the emotional, physical, and mental well-being of their care recipients.

“About my mom - She’s not the superhuman I always thought she was. She’s breakable just like the rest of us.”* (Participant 62, White)*

Many caregivers also realized the limits of their own emotional capabilities and found caregiving to be more challenging than they had imagined.

“I get overwhelmed at times and I’m afraid of their end of life.”* (Participant 68, Hispanic)*

“I need to be more patient, to think and understand what is on their mind.”* (Participant 71, Asian American)*

Change in Personality and Relationship

Over half of the caregivers (55.1%) reported a change in relationship with themselves and their loved ones throughout their caregiving journey. These changes ranged from negative (36.8%) to positive (63.2%). While most participants across all the three groups experienced an improved connection with their care recipients characterized by deeper appreciation and filial piety, some caregivers noted troubled relationships marked by increased disagreements and conflict.

“I could pay back my care and attention to my parents or for Chinese people... the best caregiver is their children.” *(Participant 66, Asian American)*

“Sometimes people do not appreciate what you do and will put others who do not help with their care at all before the one who has always done everything for them.”* (Participant 49, Hispanic)*

Knowledge of Government Policy and Norms Challenges

Less than one-third of the caregivers (30.4%) acknowledged the availability of government resources and expressed an understanding of societal and cultural norms regarding caregiving. Nearly a quarter of the respondents (23.2%) felt neglected by the government and frustrated with the inadequacy of available support and assistance. Interestingly, 14 out of the 16 respondents (87.5%) who expressed disappointment with the availability and accessibility of government support were White.

“Well, truthfully - it is deplorable. They work all their life and when they need help, ‘They make too much,’ all medications not paid for… even the insurance and healthcare providers we go to for help are burnt out.” *(Participant 30, White)*

“You have to advocate to get things done in a timely manner. You have to be persistent and follow up.” *(Participant 42, White)*

Knowledge of Programs, Services, and their Limitations

Of the 69 informal caregivers, 27 of them (39.1%) expressed their understanding of the programs and services available to support their caregiving efforts and their grievances about the challenges of navigating these resources. The majority of these respondents expressed difficulty with navigating complex systems, whether it be due to convoluted bureaucratic hoops or cultural barriers that hindered communication and understanding. The White respondents had significantly more knowledge of support programs and services than the Hispanic (*p*=0.016) and Asian American respondents (*p*=0.016).

“There are plenty of services available if you know how to access them, if you qualify, and the big if - if you have the time to figure the systems out.” *(Participant 62, White)*

Many caregivers acknowledged the availability of support programs but saw inequities in who was eligible to receive assistance, especially for minority populations and persons with disabilities.

“[I learned that there are] health inequities and discrimination against invisible disabilities.” *(Participant 4, Hispanic)*

## Discussion

Several studies [15-17] have explored the challenges and coping strategies of informal family caregivers. However, few studies [18,19] have analyzed how caregiving experiences may vary by race and ethnicity. Developing an understanding of how cultural values may shape caregiving experiences can inform the design and implementation of effective, culturally tailored interventions and policies to support caregivers and care recipients with ADRD, cancer, and other common chronic and disabling conditions. 

**Figure 2:**
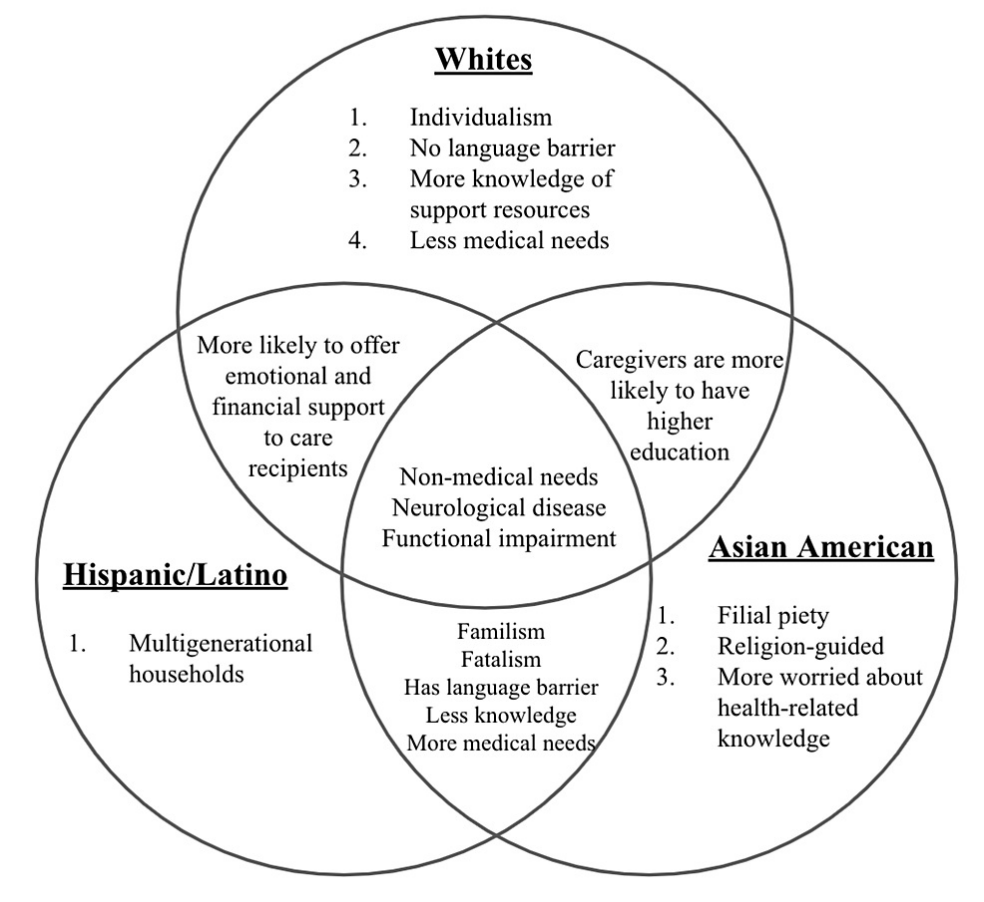
Comparison of cultural values and caregiving perspectives by race and ethnicity

The present study attempts to address this literature gap in order to design better interventions that can meet caregivers’ needs. The aim of this study is to identify potential trends in caregiving burden and coping strategies among racial and ethnic groups as an initial step in understanding possible disparities in caregiving experiences. We compared differences in the perceived burdens, changes in relationships with care recipients, and utilization of government resources and available support programs. We also explored whether the type of caregiving (e.g., financial and house chores) or health condition of care recipients (e.g., neurological vs. cardiovascular) varied by race and ethnicity. By putting their survey responses into a cultural context, Figure 2 illustrates the comparisons across the three racial groups.

Our previous study explored caregiving experiences and challenges through an analysis of the aggregate survey data, without racial and ethnic comparisons [20]. This study found that informal caregivers face significant physical and financial burdens, struggle to navigate government resources and policies, and desire more support and respite opportunities. These aggregated insights underscored the need for better interventions to enhance the well-being of caregivers and care recipients. To explore potential trends in the survey data, our present study attempts to gain more insights into factors impacting caregiving challenges through stratification by race and ethnicity. The idea that caregiving perspectives and outcomes may vary by race and ethnicity has been explored in prior caregiving studies. For example, one study from the National Health and Aging Trends Study (NHATS) and National Study of Caregiving (NSOC) found differences in care burden, psychological well-being, and self-rated health between White, Black, and Hispanic caregivers while controlling for covariates, such as age, education, and presence of primary and secondary stressors [21].

The results of the present study point toward differences in caregiving perspectives, coping strategies, needs, and outcomes among the three racial and ethnic groups surveyed. Overall, Asian American and Hispanic caregivers had greater needs and worse outcomes than White caregivers, suggesting an inversely proportional relationship between knowledge and resource needs. Because the White caregivers reported greater ease with navigating government resources, they experienced less caregiving burden compared to the Hispanic and Asian American caregivers. This trend in caregiving burden is seen across the literature.

Cultural values may shape caregiving perspectives and coping strategies

Race and ethnicity may play a role in how caregivers perceive intensity of stressors, availability of resources, and use of coping strategies [18]. One review found that cultural values influence the choice and use of coping resources (e.g., support systems and coping strategies) [19]. The sociocultural stress and coping model, outlined by Knight and Sayegh [19], postulates that the negative effects of caregiving can be mitigated by the appropriate use of coping resources, which impact emotional, physical, and mental health outcomes of both caregivers and care recipients. This model proposes that racial, ethnic, and cultural factors influence the stress and coping process, resulting in differential outcomes among caregivers of diverse backgrounds.

One cultural value that pervades caregiving research is that of familism and filial obligation [6]. Familism refers to a strong solidarity with one’s family and underscores the perspectives of many ethnic minority groups toward caregiving. Both Latino and Asian American cultures highlight the importance of the family’s responsibility to care for one another; thus, caregivers from these backgrounds tend to adopt the caregiver role more willingly and with less distress [5,6,11]. However, evidence has shown that the influence of familism can contribute to a greater sense of obligation to care for their loved ones and increased levels of avoidant rather than active coping styles. Although filial obligation beliefs are stronger among ethnic minority caregivers [6,18], White caregivers also experience a sense of familial obligation that has been linked with detrimental mental and physical health outcomes [9,22]. Because White caregivers are generally raised in a more individualistic culture, they may be more likely to perceive caregiving as a burden and disruption to their own life and personal goals [23].

Familism among Asian Americans takes the form of filial piety, a virtue that encompasses respect, care, and love for one’s parents. Adult children are expected to sacrifice their own needs for the benefit of their parents in order to fulfill their filial responsibility. Current literature has revealed a mixed relationship between filial piety and caregiver burden. One meta-analysis found that filial piety has a protective impact on caregiver burden in Eastern cultures, albeit weak [24]. One possible explanation for this positive correlation is that caregivers may associate strong filial piety with ideals of sacrifice (i.e., giving back to their parents). However, fulfillment of filial obligation may put some Asian American caregivers at a greater risk of stress and depression. In addition, sharing the burden of caregiving among family members can generate conflict and resentment due to different expectations and levels of involvement in caregiving [23].

Besides familism, another value emphasized in both Latino and Asian cultures is that of fatalism. Fatalism refers to the perception of circumstances as fate and thus outside of one’s control. It underlies coping strategies, such as attending church, faith, and spirituality. Latino and Asian American caregivers upholding the belief in fatalism are able to find comfort in their religion or spiritual practices and find more meaning and fulfillment in their caregiver role [7], which was consistent with our present study’s findings that nearly one-third of the Asian American caregivers surveyed lean on religion and prayer as ways to alleviate the burdens of caregiving. One study found that Latino caregivers are also more likely to cope through regular prayer and attending religious activities than White caregivers [11].

Cultural values may impact caregiving needs and outcomes

Numerous models have been proposed to explain how caregivers from different cultural backgrounds are differentially impacted by caregiving. Our present study found that the Asian American and Hispanic respondents had the most medical-related needs compared to the White respondents, whereas all the three groups had high non-medical-related needs. Consistent with our findings, one study of informal family caregivers in Texas for post-stroke patients found that Hispanic caregivers required more help than White caregivers for assistance with activities of daily living, such as walking, bathing, and eating [9]. Caregivers’ health status, which may decline as a direct result of caregiving, may in itself serve as an extra source of burden [7].

The values of familism, filial piety, and fatalism have been found to influence the physical, psychological, and emotional well-being of caregivers. One review found that although Asian Americans and Latinos reported the highest levels of familism, Asian Americans reported higher levels of burden and distress, and Latinos reported higher levels of depression [25]. Among both Hispanics and Asian Americans, the cultural emphasis on familism may cause caregivers to prioritize the needs of their loved ones at the expense of their own well-being, resulting in adverse mental and emotional health outcomes [19]. Similarly, although belief in fatalism can lead to greater comfort and fulfillment in one’s caregiver role, it has also been associated with adverse health outcomes. Fatalism can influence perceptions of health conditions, such as ADRD, guiding some caregivers to view dementia as a natural part of aging rather than as a disease, resulting in underutilization of support services, delayed treatment, and poorer health outcomes for care recipients [10].

Our present study found that Hispanic and Asian American caregivers face greater systemic challenges than White caregivers with navigating the Western society. This could be due to cultural and linguistic barriers. Hispanic and Asian American caregivers reported having the least information on government resources and support policies and had fewer grievances about their limitations than their White counterparts. One systematic review of Chinese-American dementia caregiver studies contends that caregiver behaviors are partly influenced by their cultural values, resulting in higher levels of unmet needs. Stigma against mental health and a lack of trust in health professionals both contributed to the disparity seen in unmet needs, despite having similar needs to caregivers in other ethnic groups [26].

Study limitations

Some limitations to the present study should be noted. First, we did not adjust for potential confounders, such as characteristics of caregivers (e.g., age, gender, income, and education level) or care recipients (e.g., severity and length of health conditions). One study exploring patterns of caregiving among Hispanic, Black, and White caregivers in Florida found that ethnic differences in caregiver resource utilization depended on an interaction of ethnicity with the caregivers’ age and education [27]. These factors may have contributed to the observed differences in our study as well.

Second, a language barrier may have limited survey respondents from understanding the questions or providing detailed answers. Several Hispanic and Asian American respondents answered with “N/A” or short responses, in contrast with more elaborate responses from White participants, which limited our content analysis. The survey was not explained or translated to Spanish. In addition, most of the Asian American respondents were Chinese, limiting the generalizability of results to other Asian American subgroups, such as Vietnamese and Korean. Third, we did not have African Americans in the sample, thus limiting our findings to only Asian Americans, non-Hispanic White, and Hispanics. Finally, because our survey was administered during the COVID-19 pandemic, the negative aspects of caregiving may have been amplified due to increased economic and psychosocial stressors during this time. 

While our survey gathered valuable insights from a diverse sample of caregivers, it is important to note that the self-reported nature of the data may introduce response bias and potential inaccuracies in the reported frequency and type of care provided. Despite limitations, our results highlight key differences and similarities in caregiving experiences by race and ethnicity and represent an important initial exploration of the potential impact of cultural factors on caregiving experiences.

## Conclusions

Informal caregivers face many mental, emotional, physical, and financial challenges in caring for their loved ones. Understanding how race and ethnicity may influence the experiences and perspectives of informal caregivers can inform the development of more effective interventions to support caregivers and care recipients from diverse cultural backgrounds. Our study found that White, Hispanic, and Asian American informal caregivers vary in their types of worries, care provided, and challenges faced. Asian American caregivers reported greater worry about the health-related knowledge of their family members than White and Hispanic caregivers. White caregivers were better at navigating government resources and caregiver support programs than Hispanic and Asian American caregivers. While cultural values, such as familism, filial piety, and fatalism, may have contributed to these differences, our results indicate the need for further research with a larger and more diverse population to draw more definitive racial and ethnic comparisons. Other factors that may have played a role include age, income, education level, and disease severity. By understanding all the factors that influence caregiving experiences, clinicians and researchers can develop more effective and culturally appropriate interventions to reduce caregiving burden and advocate for the health and well-being of informal caregivers and care recipients from all backgrounds.
